# Morphological and immunohistochemical characterization of spontaneous endometriosis in rhesus macaques (*Macaca mulatta*)

**DOI:** 10.5194/pb-4-77-2017

**Published:** 2017-04-13

**Authors:** Eva Gruber-Dujardin, Martina Bleyer, Kerstin Mätz-Rensing

**Affiliations:** Pathology Unit, German Primate Center, 37077 Göttingen, Germany

## Abstract

Several cases of spontaneous endometriosis in middle-aged to old rhesus macaques (*Macaca mulatta*) from the breeding colony of the German Primate Center were thoroughly characterized with regards to anatomical distribution and macroscopic appearance, histological differentiation and immunohistochemical profile including somatic markers, hormonal receptors, and proliferation indices. More than half of the examined animals (five of nine) were directly related to one breeding male, supporting a strong genetic predisposition. Histologically, four different types of endometriotic lesions, depending on the degree of ectopic endometrial gland and stromal differentiation (well differentiated, purely stromal, mixed differentiation, poorly differentiated), could be constantly identified within all animals. Immunohistochemistry (IHC) of cytokeratin (CK), vimentin, smooth muscle actin (SMA), desmin, estrogen (ER), and progesterone (PR) receptors as well as of the nuclear proteins Ki67 and p53 revealed varying staining patterns in the four different types of endometriosis differentiation and compared to normal endometrium. Purely stromal, mixed, or poorly differentiated lesions, especially, showed additional cytokeratin-positive stromal cells, whereas epithelial cells of endometriosis with mixed or poor differentiation increasingly expressed mesenchymal markers (vimentin, SMA). Hormonal receptor and Ki67 expression in well-differentiated endometriotic lesions mostly reflected that of normal endometrial tissue according to the cyclic phase of the animal, while the expression gradually diminished with decreasing grade of differentiation. However, increased nuclear accumulations of p53 antigen could only be continuously detected in epithelial cells of mixed or poorly differentiated endometriosis. Altogether, these findings support the pathogenetic theory of coelomic metaplasia, since the expression profiles of somatic markers in less differentiated forms closely resembled that of mesothelial cells. Thus, the four different histological types of endometriosis might display subsequent grades of differentiation in the course of time, with poorly differentiated types representing newly formed, immature lesions and well-differentiated types being older, fully differentiated forms, rather than being the outcome of dedifferentiation processes.

## 1 Introduction

Endometriosis is a common chronic gynaecological disorder in women, with a prevalence of at least 10 % (Giudice and Kao, 2004; Brosens et al., 2016), which also occurs in several Old World primate species that have a menstrual cycle (Assaf and Miller, 2012; Barrier et al., 2007; Dick et al., 2003; Wilkinson et al., 2008; Zondervan et al., 2004). It is defined by the presence of ectopic endometrial glandular and stromal tissue outside the uterine cavity, accompanied by nonspecific clinical symptoms, like dysmenorrhea, pelvic pain, or infertility, and has a highly variable manifestation concerning the anatomical distribution as well as macroscopic and histological appearance (Abrao et al., 2003; Adamson, 2011; Mattison et al., 2007; Mounsey et al., 2006). Although extensive literature has been published on possible pathogenetic mechanisms and promoting factors (Brosens et al., 2016; Brosens and Brosens, 2000b; Defrere et al., 2008a; Gotte et al., 2011; Kobayashi, 2000; Sasson and Taylor, 2008; Schindler, 2007; Toki and Nakayama, 2000; Young et al., 2013) and many animal models in primate as well as non-primate species have been more or less successfully introduced (Defrere et al., 2008b; Dinulescu et al., 2005; Einspanier et al., 2006; Fazleabas et al., 2002; Gashaw et al., 2006; Grummer, 2006; Rier et al., 1993), the etiology of endometriosis remains obscure. The pathogenetic mechanism most widely accepted as a prerequisite for the development of endometriotic lesions is retrograde menstruation that is the distribution and implantation of refluxed endometrial material within the peritoneal cavity (Sampson, 1927; for review: Brosens and Brosens, 2000b). This theory is supported by the frequent involvement of pelvic organs and the fact that this disease almost exclusively develops in women or female nonhuman primates that are menstruating. It has also been demonstrated that forced induction of retrograde menstruation in a baboon model contributed to the development of the disease phenotype (Braundmeier and Fazleabas, 2009). However, the phenomenon of retrograde menstruation occurs in the majority of women, of which only a small percentage develop endometriosis (D'Hooghe and Debrock, 2002). Therefore, further pathogenesis theories, such as those of coelomic metaplasia or embryonic rest, have been suggested. Coelomic metaplasia means that mesothelial cells generally have the potential to differentiate into functional endometriotic tissue (Gruenwald, 1942), triggered by an appropriate stimulus, e.g., certain humoral factors in menstrual fluid or steroid hormones (Young et al., 2013; Giudice and Kao, 2004). The embryonic rest theory proposes that, at puberty, there is an activation of cells of Muellerian duct origin at various sites within the pelvic cavity (Batt et al., 1990). Both theories are supported by reports on prepubescent and adolescent girls (Brosens et al., 2016) and even rarely men undergoing hormone treatment diagnosed with endometriosis (Pinkert et al., 1979; Schrodt et al., 1980). In addition, several supplementary conditions are assumed to contribute to the development of the disease, including genetic (Borghese et al., 2017; Thomas and Campbell, 2000; Zondervan et al., 2004), environmental (e.g., iron and dioxin; Lousse et al., 2009; Rier et al., 1993), or immune-mediated factors (Giudice and Kao, 2004). Increasing numbers of publications also point to an important role of stem or progenitor cells, respectively, in the pathogenesis of endometriosis (Forte et al., 2014; Forte et al., 2009; Gotte et al., 2011; Matthai et al., 2006; Sasson and Taylor, 2008).

Another striking feature of endometriosis is its frequently reported association with malignancies. It is proposed that certain aspects of this disease are similar to those of malignant neoplasia, e.g., uncontrolled proliferation, infiltrative growth, and lymphogenic or vascular spread, and, thus, it might possess precancerous potential (Kobayashi, 2000; Mandai et al., 2009; Nezhat et al., 2008; Van Patten et al., 2010; Yoshikawa et al., 2000). But the ability to induce malignant transformation of other tissues, e.g., by persistent oxidative stress from endometriosis-dependent recurrent hemorrhage, is also considered (Higashiura et al., 2012; Nishida et al., 2000; Tanase et al., 2013). In summary, there are many different facets of this disorder, with some of them not fully understood yet, painting a heterogeneous and very complex picture.

Spontaneous endometriosis in nonhuman primates often remains unnoticed until the animal either suddenly dies from the fatal course of the disease or deceases for other reasons (Dick et al., 2003; Mattison et al., 2007; Zondervan et al., 2004). Also in women, an early, noninvasive diagnosis of endometriosis is challenging due to nonspecific symptoms and heterogeneity of the disease pattern (Mehedintu et al., 2014). Hence, clinical staging and prognosis is often referred to localization and histological differentiation, and various classification systems have been introduced to predict clinical outcome in patients (Adamson, 2011). But, since the preferred diagnostic method for endometriosis is still the surgical endoscopic inspection of the abdominal cavity (mainly pelvic organs) with histological evaluation of tissue biopsies, early, very small, or disseminated lesions, which might have a divergent gross or histological appearance, are easily overlooked (Mounsey et al., 2006). Accordingly, current studies have proven that there is a poor to no correlation between the extent of the diagnosed disease and its clinical symptoms or therapeutic success (Mehedintu et al., 2014).

In order to receive a comprehensive overview of the heterogeneous macroscopic and associated histological features in nonhuman primates and to gain further insights into its pathogenesis, a thorough and systematic morphological characterization of spontaneous endometriosis in a large group of rhesus macaques from the breeding colony of the German Primate Center has been conducted, with special focus on varying histological grades of differentiation and the corresponding immunohistochemical profile of several somatic, hormonal, and proliferation markers.

## 2 Materials and methods

### 2.1 Animals

All rhesus macaques (*Macaca mulatta*) included in this study came from the breeding colony of the German Primate Center in Göttingen, Germany, and were kept according to the regulations of the European Parliament and the Council Directive on the protection of animals used for scientific purposes (2010/63/EU), the National Institutes of Health Guide for the Care and Use of Laboratory Animals (2010), and the applicable German Animal Protection Law (“Tierschutzgesetz/Tierschutzversuchstierverordnung”). None of the investigated animals had ever been subject to any experimental intervention. They were housed within metal and concrete indoor–outdoor facilities in large matrilineal groups of 25 up to 100 animals with one breeding male per group and fed commercial monkey diets supplemented with fresh fruits and vegetables as well as curd mash, enriched with vitamins and minerals. Water was available ad libitum. All breeding macaques of the facility are annually screened for *Macacine herpesvirus* 1 (B virus), tuberculosis, and echinococcosis. Out of 154 adult female rhesus macaques from the breeding colony that were necropsied at the Pathology Unit of the German Primate Center over a period of 10 years (from 2001 to 2011), 9 cases had recorded spontaneous endometriosis, mainly confirmed by histopathology of post mortem tissue. All of them were retrospectively evaluated for pedigree, medical, surgical and reproductive history, and cause of death as well as macroscopic appearance and distribution of endometriotic lesions at necropsy, while eight of them were additionally reinvestigated by histopathological and immunohistochemical analysis.

### 2.2 Histology and immunohistochemistry

During necropsy, representative tissue specimens of all major organs as well as additional tissue with grossly visible changes had been collected from each animal and were fixed in 10 % neutral buffered formalin, embedded in paraffin, and sectioned at approximately 4 µm before being routinely stained with hematoxylin–eosin (HE) stain. Occasionally, special stains, including the periodic acid–Schiff (PAS)-reaction and Masson's trichrome stain, were used to further characterize particular features of epithelial and stromal cells as well as the intercellular matrix. Immunohistochemistry (IHC) was performed on all paraffin-embedded sections that contained endometriotic lesions and/or normal endometrium, using the avidin–biotin peroxidase method with an automated immunohistochemical staining system (Ventana Discovery XT). Antibodies were directed against cytokeratin (MNF 116, broad spectrum CK, 1:100 monoclonal mouse anti-human, Dako), vimentin (V9, 1:100, monoclonal mouse anti-human, Dako), smooth muscle actin (SMA; 1A4, 1:400, monoclonal mouse anti-human, Dako), desmin (D 33, 1:100, monoclonal mouse anti-human, Dako), Von Willebrand factor (vWF; Factor VII-related antigen, 1:25, Dako), estrogen receptor (ER; alpha 6F11, 1:10, monoclonal mouse, Lab Vision), progesterone receptor (PR; PgR636, 1:50, monoclonal mouse anti-human, Dako), nuclear protein Ki67 (MIB-1, 1:50, monoclonal mouse anti-human, Dako), and p53 protein (DO 7, 1:50, monoclonal mouse anti-human, Dako). In all immunohistochemical staining procedures, diaminobenzidine (DAB) was used as the chromogen and slides were counterstained with hematoxylin.

### 2.3 Histological classification

For morphologic evaluation, a histological classification was applied, which is also used in women, comprising four different forms of endometriosis based on the degree of differentiation (Abrao et al., 2003; Kamergorodsky et al., 2009; Porto et al., 2015) described as follows. The first is a well-differentiated, glandular form with presence of surface epithelium or epithelium with glandular to cystic formations. The cells are indistinguishable from those of normal endometrium during different phases of the menstrual cycle (proliferative/follicular, secretory/luteal, menstrual, and regenerative; Van Esch et al., 2008). The second is a pure stromal form of endometriosis without any surface or glandular epithelium. The stroma also mainly resembles that of normal endometrium during different phases of the menstrual cycle. The third is a glandular pattern of mixed differentiation, with the epithelium being composed of both cylindric to columnar endometrial-like cells, low cuboidal to flattened cells, and undifferentiated cells, and also sometimes cells with other histological Muellerian patterns (serous or mucinous cells). And finally, the fourth is a poorly differentiated form that is characterized by a solely undifferentiated glandular pattern in which the surface epithelium or glandular/cystic formations are exclusively lined by low cuboidal to flattened, mesothelial-like cells or appear as small epithelial nests or islands.

**Table 1:** Case details of nine female rhesus macaques of the DPZ (German Primate Center) breeding colony with spontaneous endometriosis.

No.	ID	Age	Weight	Cause of death;	Mother	Father	Offspring	Medical history
		(years)		gross findings			(last birth)	
1	1764${}^{a}$	19	8879 g	Severe hemoperitoneum;	1568	1585${}^{a}$	6	No history of abortion
				widespread fibrous			(8 years before)	or abdominal surgery
				peritoneal adhesions				
2	1712	20.5	6088 g	Septicemia;	1563	1585${}^{a}$	7	No history
				multiple abscesses, fibrous			(9 years before)	of abortion or
				adhesions of pelvic organs				abdominal surgery
3	2056	10.5	7617 g	Euthanasia	1764${}^{a}$	1619	2	No history
				(abdominal mass);			(4 years before)	of abortion or
				fibrous adhesions of pelvic				abdominal surgery
				organs, large ovarian				
				cyst, hydronephrosis				
4	9304	24	8120 g	Euthanasia	n.a.	n.a.	n.a.	No history
				(severe weakening);				of abortion
				multiple intraabdominal masses				or abdominal surgery
				and fibrous adhesions,				
				large intrauterine mass				
5	1575	17.5	9828g	Euthanasia	1569	n.a.	7	Last offspring:
				(abdominal mass);			(4 years before)	stillbirth; no history
				thickened uterus and ovaries				of abdominal surgery
				within large cystic mass,				
				fibrous adhesions of				
				pelvic organs, hydronephrosis				
6	10 984	n.a.	7770 g	Euthanasia	n.a.	n.a.	n.a.	2 years ago: ret. plac.
				(intrauterine mass);				premature delivery;
				thickened uterus and				3 month ago:
				ovaries within large cystic				abdominal surgery
				mass and fibrous adhesions				
7	1518	19.5	7968 g	Euthanasia	1514	n.a.	4	Last offspring:
				(severe weakening);			(11 years before)	stillbirth; no history
				fibrous adhesions of				of abdominal surgery
				pelvic organs,				
				large ovarian cyst				
8	1594${}^{b}$	25	5067 g	Septicemia;	1559	1585${}^{a}$	8	One stillbirth;
				fibrous adhesions of pelvic			(5 years before)	no history of
				organs with multiple				abdominal surgery
				abscesses and rectal perforation				
9	1595${}^{b}$	23	6562 g	Age-related weakness;	1559	1585${}^{a}$	7	No history
				thickened uterus with			(4 years before)	of abortion or
				multiple transmural				abdominal surgery
				hemorrhagic cysts				

${}^{a}$ Animals are directly related. ${}^{b}$ Twins; n.a.: not announced; ret. plac.: retained placenta.

### 2.4 Data analysis

Eight rhesus macaques (no. 1–8) were systematically scanned for the presence and distribution of the described histological types of endometriosis within all collected representative histological HE sections. Every tissue/organ involved together with the corresponding grade and growth pattern (expansile vs. infiltrative) was recorded. Immunohistochemical expression patterns of normal endometrium as well as epithelial and stromal components of different histological types of endometriosis were evaluated semi-quantitatively according to Ferreira et al. (2012). Staining for cytokeratin, vimentin, SMA, desmin, and vWF, was, respectively, scored in five categories: negative (-) with < 5 % of cells stained, sparse ((+)) with 5 to 25 % of cells stained, mild (+) with 25 to 50 % of cells stained, moderate or heterogeneous (++) with 50 to 75 % of cells stained, and strong (+++) with > 75 % of cells stained. For estrogen and progesterone receptors, an “Allred score” was assigned (Allred et al., 1998; Harvey et al., 1999), consisting of a proportion score, representing the estimated proportion of positive-staining cells (0, none; 1 < 1/100; 2, 2/100–1/10; 3, 1/10–1/3; 4, 1/3–2/3; and 5, > 2/3), and an intensity score, representing the average staining intensity (0, none; 1, weak; 2, intermediate; and 3, strong). The proportion and intensity scores were finally added to obtain a total hormonal receptor score ranging from 0 to 8. The proliferation index (PI) was determined by calculating the percentage of Ki67-positive nuclei within at least 500 cells. This was automatically estimated from digital slides (slide scanner Aperio CS 2, Leica, Germany) within representative regions in highly positive areas without inflammation and/or necrosis, using an adapted nuclear algorithm (Aperio ImageScope Version 12.3.2.5030, Leica Biosystems). The p53 staining was considered positive if more than 10 % of cells exhibited positive nuclear staining in 10 high-power fields (HPF, 40x magnification), independent of staining intensity. For every IHC antibody, semi-quantitative scores or PIs, respectively, were recorded in an excel spreadsheet (Microsoft Office, 2010), and mean values were calculated from the eight animals analyzed. For calculation of the five semi-quantitative categories, the scores (-, (+), +, ++, +++) were transferred into corresponding numerical data from 0 to 4.

## 3 Results

### 3.1 Clinical history and gross lesions

Case details of all female rhesus macaques with spontaneous endometriosis, including age, cause of death, main macroscopic findings, pedigree, breeding, and medical history, are summarized in Table 1. All affected animals were middle-aged to old, ranging from 10.5 to 25 years, and, except for one, none of them had a history of abdominal surgery. They were either euthanized for different reasons, like the presence of an inoperable intraabdominal mass or severe weakening, or died spontaneously due to severe hemoperitoneum, septicemia, or age-related weakness. More than half of the monkeys (five of nine) were directly related to one breeding male (1585), either as a daughter or granddaughter, with two of them being twins (nos. 8 and 9). As far as is known, all had multiple offspring with two to eight infants, but the last births had always been long ago (4–11 years before death). Four cases had a history of stillbirth or premature delivery.

**Table 2:** Organ distribution and growth pattern of different histological types of spontaneous endometriosis in eight rhesus macaques from the DPZ breeding colony.

	Well differentiated		Pure stromal		Mixed		Poorly differentiated	
No.	expansile	infiltrative	expansile	infiltrative	expansile	infiltrative	expansile	infiltrative
1	liver	mesentery	uterus	mesentery	uterus	omentum	colon	mesentery
		colon	colon		liver	stomach		
		uterus	diaphragm		spleen	mesentery		
		urinary bladder			colon	colon		
		diaphragm			diaphragm	diaphragm		
2	urinary	mesometrium	urinary		urinary	uterus		
	bladder	ovary/tube	bladder		bladder	mesometrium		
						ovary/tube		
3		mesometrium	mesometrium	mesometrium		mesometrium		mesometrium
			(decidualized)	ovary/cyst		ovary/cyst		ovary/cyst
				ureter				
4	uterus	uterus		omentum	small	omentum		
	(endometrial	ovary/tube			intestine	ovary/tube		
	hyperplasia)							
5	uterus	uterus	colon	mesometrium	colon	mesometrium		mesometrium
		(adenomyosis)			uterus			
6	uterus	uterus	uterus	mesometrium/	urinary	mesometrium		mesometrium
		(adenomyosis)	urinary	mesovarium	bladder		mesovarium	
		mesometrium	bladder		mesometrium			
		mesentery	mesometrium		(decidualized)			
		diaphragm	(decidualized)					
7	uterus	mesovarium/	ovary/cyst	tube	ovary/cyst	colon		tube
	mesovarium/tube	tube	tube		tube	mesometrium		
						tube		
8	uterus	urinary	uterus		uterus	colon		mesentery
	colon	bladder				mesentery		uterus
								mesometrium

**Figure 1:**
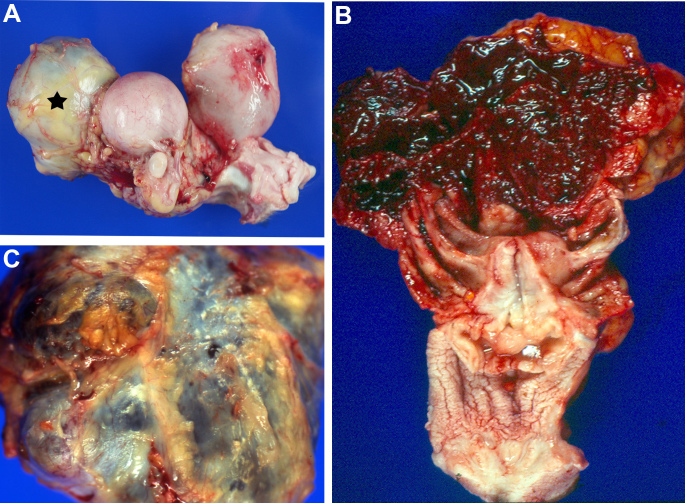
Representative macroscopic pictures of spontaneous endometriosis in rhesus macaques (*Macaca mulatta*): **(a)** ovarian cyst (star) attached to the uterus (middle); note the normal size of the right ovary and red serosal plaques on the urinary bladder, indicative of endometriotic lesions (animal no. 3); **(b)** large multicystic mass with bloody contents comprising the entire uterus and adnexa (animal no. 5); **(c)** characteristic yellow-white to red plaque formation on serosal surface of a large multicystic intraabdominal mass (animal no. 6).

**Figure 2:**
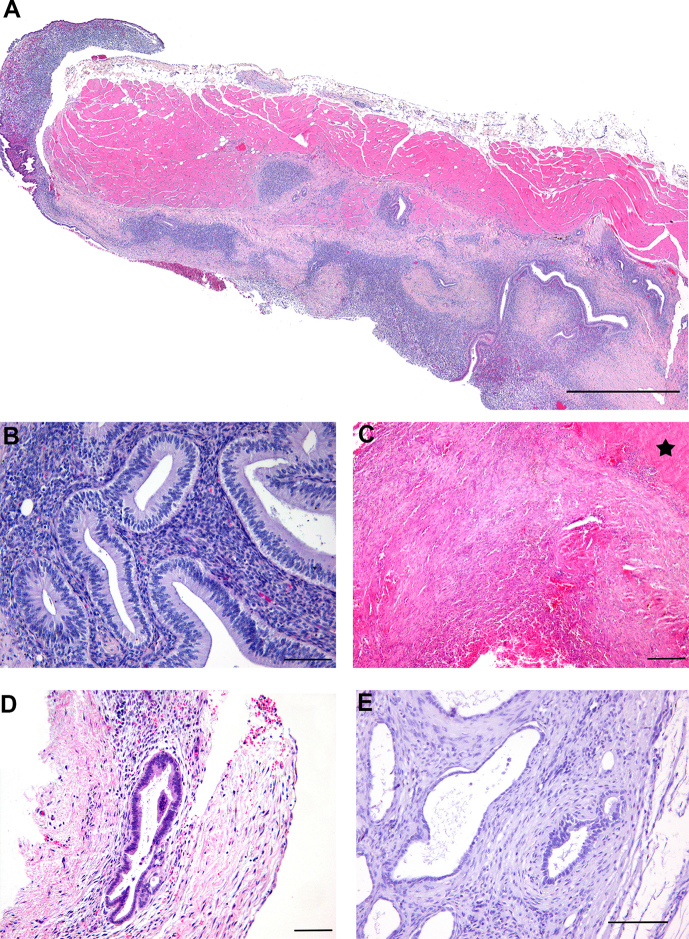
Growth pattern and histological grades of differentiation in endometriotic lesions of rhesus macaques (*Macaca mulatta*): **(a)** diaphragm muscle with widespread expansile and infiltrative endometriotic lesions on the peritoneal surface of animal no. 1, scale bar 1 mm, HE stain; **(b)** well-differentiated endometriosis with pseudostratified columnar glandular epithelium and cell-rich stroma in animal no. 6, scale bar 100 µm, HE stain; **(c)** pure stromal endometriosis without glandular formations on serosal surface of the colon (star) in animal no. 5, scale bar 200 µm, HE stain; **(d)** mixed-type endometriosis with poor and well-differentiated glandular epithelium in animal no. 1, scale bar 200 µm, HE stain; **(e)** poorly differentiated endometriosis with low cuboidal to flattened glandular epithelium in animal no. 7, scale bar 100 µm, HE stain.

Representative macroscopic changes during necropsy are depicted in Fig. 1. The most common finding, present in eight of nine animals, was fibrous peritoneal adhesion of varying extent but always comprising at least the uterus, ovaries with adnexa, urinary bladder, and terminal parts of the colon (Fig. 1a). In five cases, additional cystic to multicystic masses were found, either on the ovary (Fig. 1a) or incorporating the entire uterus with adnexa (Fig. 1b). The cysts contained serohemorrhagic to bloody fluid and were often lined by polypoid to nodular projections. The serosal surface of affected organs generally showed multifocal to coalescing whitish-yellow to red, firm to soft plaques, strands or nodules of varying size, ranging from few millimeters to several centimeters (Fig. 1a, c). In all animals, those lesions were present on the uterus, ovaries and ovarian tubes, or associated masses, respectively. Further distribution on the mesometrium, urinary bladder, and colon, with respective mesenterial tissue, was present in most cases. Two cases (nos. 1 and 6) also showed lesions on the peritoneal surface of the diaphragm (Fig. 2a), with additional involvement of the liver, spleen, and stomach in animal no. 1. Another case (no. 4) had accordant changes on the small intestine. Moreover, three monkeys (nos. 5, 6, and 9) revealed remarkable thickening of the uterine wall with multiple transmural hemorrhagic cysts of varying size consistent with adenomyosis. Case no. 4 had a large, poorly circumscribed, soft, intrauterine mass emanating from the endometrium, with a variegated beige to red cut surface and multifocal small hemorrhages indicative of endometrial hyperplasia. Additional macroscopic findings related to endometriosis and representing the cause of death in three animals were severe hemoperitoneum (no. 1) or suppurative peritonitis with multiple abscesses and septicemia (no. 2 and 8), accompanied by rectal perforation in case 8. Furthermore, hydronephrosis due to ureter compression was detected in two animals (no. 3 and 5).

### 3.2 Histology

**Figure 3:**
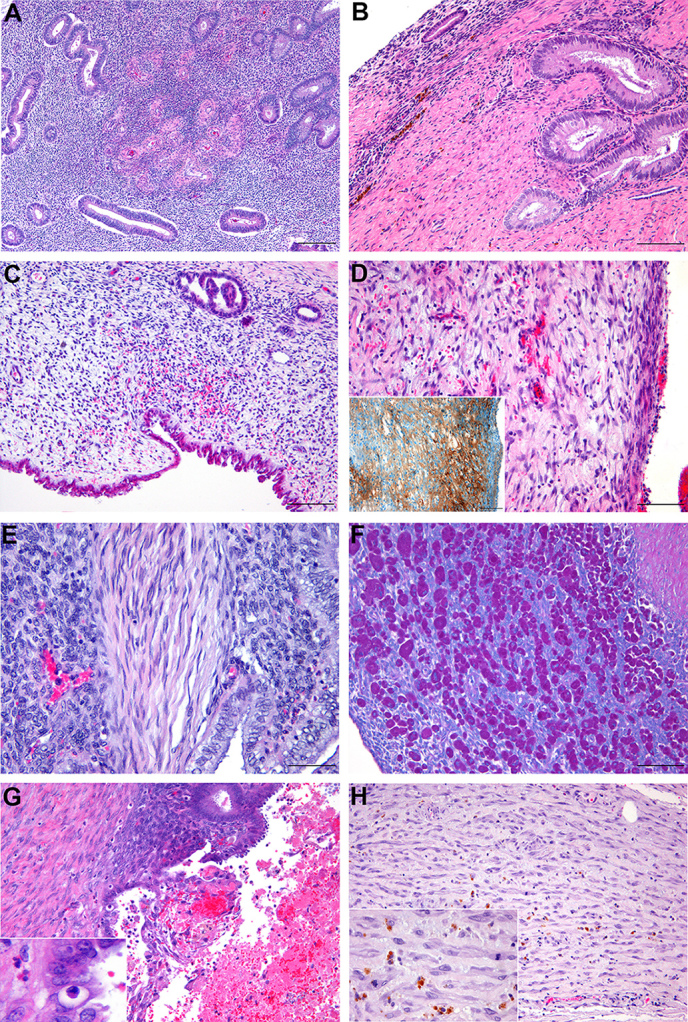
Histological variation of endometriotic lesions in rhesus macaques (*Macaca mulatta*): **(a)** well-differentiated cell-rich endometriotic stroma with formation of spiral arterioles within mesometrium of animal no. 3, scale bar 200 µm, HE stain; **(b)** collagenous (fibrotic) stroma with well-differentiated endometriotic glands on the urinary bladder of animal no. 2, scale bar 100 µm, HE stain; **(c)** loosely arranged (myxoid) endometriotic stroma cells with mild interstitial hemorrhage and glandular formations of mixed differentiation on the uterine serosal surface of animal no. 1, scale bar 100 µm, HE stain; **(d)** endometriotic stroma with distinct sprouting of fibroangioblasts, capillary formation and mild interstitial hemorrhage on the colonic serosal surface of animal no. 1, scale bar 100 µm, HE stain (inset showing IHC vWF); **(e)** smooth muscle cells within well-differentiated endometriotic stroma in the mesentery of animal no. 1, scale bar 50 µm, HE stain; **(f)** numerous decidual cells (“deciduosis”) within endometriotic stroma on the serosal surface of the urinary bladder in animal no. 6, scale bar 100 µm, PAS reaction; **(g)** endometriotic lesion with epithelial desquamation, fibrino-hemorrhagic debris, and mild stromal inflammatory cell infiltrates indicative of menstrual activity in mesometrium of animal no. 3, scale bar 100 µm, HE stain (inset showing characteristic granular leucocytes); **(h)** pleomorphic spindle cell proliferation within collagenous matrix and hemosiderin deposits adjacent to endometriotic lesions within the mesometrium of animal no. 3, scale bar 100 µm, HE stain (inset showing two mitotic figures, scale bar 20 µm).

As shown in Table 2, all different histological types of endometriosis could be detected in almost every animal, besides animal no. 2 and 4, which did not reveal poorly differentiated lesions. Within each of the four types, the endometriotic epithelium and stromal tissue either grew expansively on serosal surfaces or infiltrated the underlying tissue (Fig. 2a), apparently independent of the organ involved, though infiltration was especially pronounced in poorly differentiated mesenterial lesions. In well-differentiated endometriosis, the surface and glandular formations were lined by simple to pseudostratified columnar epithelial cells (Fig. 2b) often showing cilia or apical blebs with PAS-positive secretion, corresponding to the epithelial morphology of normal endometrium and depending on the menstrual cycle of the animal. In less differentiated areas, surface and glandular epithelium was simple cuboidal to flattened (Fig. 2d, e), sometimes resembling mesothelial or even endothelial cells. The stromal component appeared very heterogeneous (Fig. 3a–e). Although well-differentiated forms were generally characterized by a typical, highly cellular, and matrix-poor endometrial stroma sometimes accompanied by distinct spiral arterioles (Fig. 3a), many other forms with fibrotic, myxoid, fibroangioblastic, or myogenic phenotypes could be identified in all four histological types. In two animals (no. 3 and 6), decidualization of endometriotic stroma was present to varying degrees, characterized by intermingled large, polygonal to epithelioid, and sometimes binucleated cells with abundant finely granular, eosinophilic, and PAS-positive cytoplasm consistent with decidual cells (Fig. 3f). Cystic structures within endometriotic lesions were regularly encountered, either resulting from dilated glandular formations with serous to mucinous contents due to excessive secretion or, as blood-filled cavities, often accompanied by desquamation of glandular epithelium, stromal inflammatory cell infiltrates, such as characteristic granular leucocytes, and necrotic debris in terms of menstrual activity (Fig. 3g). Also interstitial hemorrhage of different degrees was frequently observed, often with advanced chronicity reflected by extracellular accumulations of hemosiderin pigment (Fig. 3g). Furthermore, two monkeys (no. 6 and 3) revealed focally extensive, poorly circumscribed areas of extensive spindle cell proliferation within the uterine wall or adjacent mesentery, respectively. Within the myometrium of case no. 6, large, plump spindle cells were arranged in fascicles or interwoven bundles, predominantly separated by a fine fibrovascular stroma with moderate amounts of fibrillar eosinophilic and sometimes vacuolated cytoplasm and central oval to cigar shaped, vesiculated nuclei and prominent nucleoli. Cells and nuclei were fairly uniform with absent mitosis suggesting benign neoplasia of smooth muscle tissue (leiomyoma). In animal no. 3, spindle cell proliferations located in the mesometrium were rather pleomorphic, often embedded in moderate amounts of light eosinophilic, fibrillary to vacuolated (collagenous) matrix material with multifocal hemosiderin deposits and revealed an increased mitotic activity (1-2/HPF) indicating mesenchymal neoplasia of low grade malignancy (Fig. 3g). The intrauterine mass found in animal no. 4 was histologically characterized by an expansile, non-encapsulated proliferation of well-differentiated glandular and stromal endometrial tissue with multifocal cystic glandular ectasia and mild to moderate interstitial hemorrhage of varying chronicity, consistent with nodular endometrial hyperplasia.

**Table 3:** Immunohistochemical staining pattern of somatic markers in normal endometrium and different histological types of endometriotic lesions from eight rhesus macaques with spontaneous endometriosis.

	Antibody				
Tissue	Cytokeratin	Vimentin	SMA	Desmin	vWF
Normal endometrium					
Epithelial cells	+++	(+)	-	-	-
Stromal cells	-	++	(+)	-	+
*Endometriosis*					
Well differentiated					
Epithelial cells	+++	(+)	-	-	-
Stromal cells	-	+++	+	-	+
Pure stromal					
Stromal cells	+	+++	+	+	+
Mixed					
Epithelial cells	+++	+	(+)	-	-
Stromal cells	(+)	+++	+	(+)	+
Poorly differentiated					
Epithelial cells	+++	++	(+)	(+)	-
Stromal cells	+	++	++	+	+

- negative (< 5 % positive cells). (+) sparse (5–25 % positive cells). + mild (25–50 % positive cells). ++ moderate or heterogeneous (50–75 % positive cells). +++ strong (> 75 % positive cells).

**Table 4:** Immunohistochemical staining pattern of estrogen (ER) and progesterone receptors (PR) as well as nuclear proteins Ki67 and p53 in normal endometrium and different histological types of endometriotic lesions from eight rhesus macaques with spontaneous endometriosis.

Tissue	Antibody			
	ER	PR	Ki67	p53
Normal endometrium				
Epithelial cells	6	7	40 %	-
Stromal cells	5	6	10 %	-
*Endometriosis*				
Well differentiated				
Epithelial cells	6	7	34 %	-
Stromal cells	5	6	13 %	-
Pure stromal				
Stromal cells	4	6	23 %	±
Mixed				
Epithelial cells	5	4	23 %	+
Stromal cells	5	6	15 %	±
Poorly differentiated				
Epithelial cells	3	2	17 %	+
Stromal cells	4	5	9 %	±

Hormonal (Allred) receptor score: 0–8, sum of quotient (0–5) and intensity (0–3); Ki67 proliferation index (PI): positive nuclei/at least 500 cell counts in highly positive areas; P53: + (positive) if > 10 % of all nuclei stain positive in 10HPF (regardless of staining intensity).

**Figure 4:**
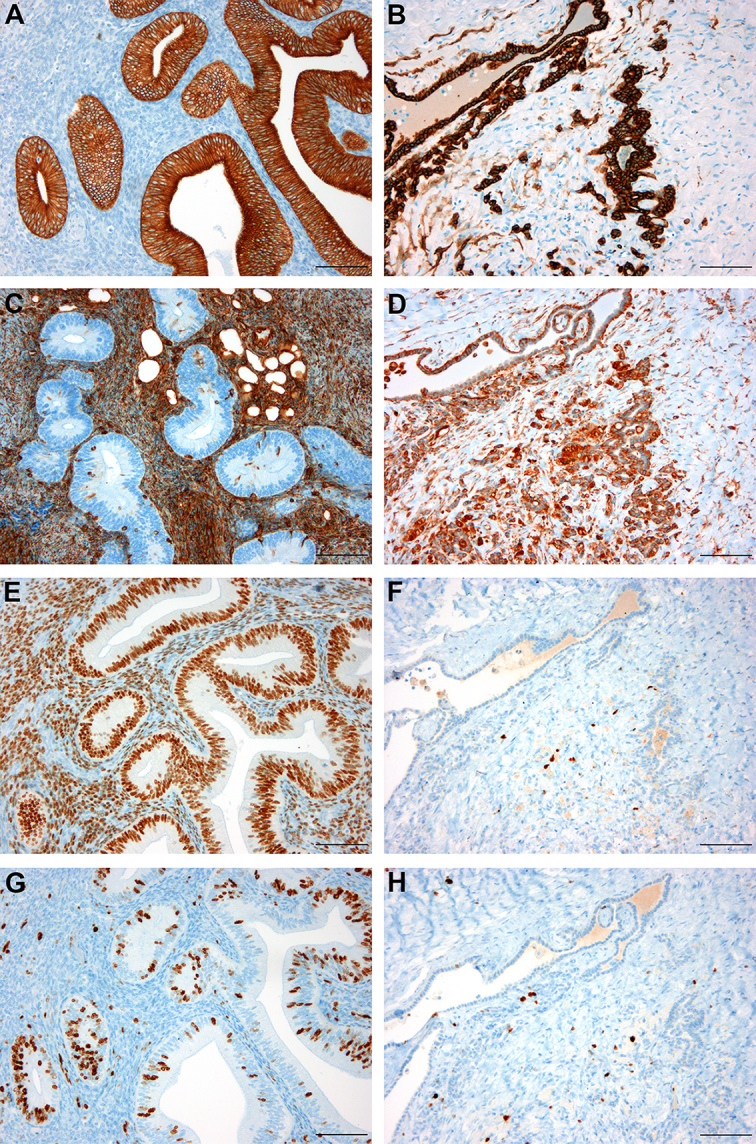
Comparison of representative immunohistochemical staining patterns between well- and poorly differentiated endometriosis: IHC for cytokeratin exclusively stains epithelial cells in well-differentiated forms **(a)** compared to additional staining of several stromal cells in poorly differentiated endometriosis **(b)**; IHC for vimentin reveals strong staining of stromal cells with only sparse positive epithelial cells within well-differentiated endometriosis **(c)** compared to distinct epithelial staining in poorly differentiated forms **(d)**; IHC for PR intensely stains stromal and epithelial cells with slightly pronounced epithelial signals in well-differentiated endometriosis **(e)** and only sparsely disseminated positive stromal cells in poorly differentiated endometriosis **(f)**; IHC for Ki67 shows numerous positive cells predominantly within the epithelium of well-differentiated endometriosis **(g)** compared to few positive stromal cells and virtually no epithelial staining in poorly differentiated forms **(h)**; hematoxylin counterstain, scale bars 100 µm.

### 3.3 Immunohistochemistry

Epithelial and stromal immunohistochemical staining patterns of the four different histological types of endometriosis and normal macaque endometrium are summarized in Tables 3 and 4. While in normal endometrial tissue, as well as well-differentiated endometriosis, glandular and surface epithelium exclusively stained positive for cytokeratin with negative stromal cells, the other types showed a gradual increase of cytokeratin-positive stromal cells with decline in differentiation (Fig. a, b). The IHC staining profile for vimentin exhibited contrary results, with virtually negative epithelial cells and intensely positive stromal cells in normal endometrium and well-differentiated endometriosis and progressive epithelial staining in mixed and poorly differentiated endometriosis (Fig. 4c, d). A similar trend, but to a lesser degree, could be observed with myogenic markers (SMA and desmin), though desmin staining was completely absent in normal endometrium or well-differentiated endometriosis, respectively. No obvious differences in staining pattern or intensity could be found with the vascular marker vWF, both between different types of endometriosis and also compared to normal endometrium. The hormonal receptor scores for ER and PR were comparatively high in normal endometrium and well-differentiated endometriosis (Fig. 4e), with slight emphasis on epithelial cells and conspicuous variation between individual animals. In less differentiated endometriosis, decreasing expression of hormonal receptors, especially within epithelial cells, could be observed (Fig. 4f). Additionally, PIs, demonstrated by the percentage of Ki67-positive cells, were almost twice as high in the epithelium of normal endometrium (40 %) and in well-differentiated endometriosis (34 %) compared to that of mixed (23 %) or poor (17 %) differentiation (Fig. 4g, h). However, this tendency was not discernible in stromal cells, with overall less Ki67 staining ranging from 9 to 23 % and highest PIs in pure stromal endometriosis. Nuclear staining of p53 was constantly positive in epithelial cells of endometriosis with mixed and poor differentiation, while the corresponding stroma and pure stromal endometriosis showed variable results. Well-differentiated endometriosis as well as normal endometrium were always negative for p53. The described spindle cell proliferation of animal no. 3 revealed positive nuclear staining for p53, together with a comparatively high PI (38 %) and intense IHC staining for CK, vimentin, SMA, and desmin, but only little to no expression of hormonal receptors. Within the same animal, also sparsely dispersed single cells positive for CK, ER and PR, respectively, could be detected within medullary sinuses of lymphonodular tissue.

## 4 Discussion

The results of this study provide a comprehensive overview of the clinical, macroscopic, and microscopic manifestations of spontaneous endometriosis in rhesus macaques (*Macaca mulatta*), emphasizing important aspects of diagnostic relevance and possible pathogenetic implications. From the breeding colony of the German Primate Center, nine rhesus macaques presented with endometriosis over a period of 10 years. Related to the overall 154 adult female breeding rhesus macaques that were necropsied at the Pathology Unit during that time span, this corresponds to an incidence of about 6 %. This is rather low, compared to the reported, highly variable incidences of spontaneous endometriosis in other captive rhesus macaque colonies ranging from 0 % to 45 % depending on the age and background of the animals investigated (Coe et al., 1998; Mattison et al., 2007). Generally, it is assumed that incidences of endometriosis in nonhuman primates used for research purposes are likely underestimated, since many mild or incidental cases that occur within experimental studies remain undetected or unreported (Mattison et al., 2007). In addition, dysmenorrhoea, defined as pronounced pain during menstruation, is one of the first clinical symptoms of endometriosis and often difficult to recognize in monkeys. This impedes an early intra vitam diagnosis of the disease, so that in most studies only rather severe or fatal cases are taken into account (MacKenzie and Casey, 1975). Accordingly, all rhesus macaques from this study were of older age and exclusively presented with advanced forms of endometriosis at necropsy, even though this was often not the primarily suspected diagnosis. This suggests that younger females from the population with milder forms of the disease might have been undetected. In this respect, breeding populations with a high incidence of endometriosis should be monitored by careful records of menstruation history and routine digital rectal examination in order to identify some early changes (Fanton et al., 1986). A well-known risk factor increasing the incidence of spontaneous endometriosis in captive nonhuman primates is abdominal surgery, including caesarean sections, ovarian follicle aspiration, and embryo transfers (Coe et al., 1998; Dick et al., 2003). Since all examined animals, except one, did not have a history of surgical intervention, this had not been a crucial factor for the development of the disease in the present study. In contrast, a familial link for endometriosis with increased risk for first-degree relatives, as reported for both nonhuman primates (Zondervan et al., 2004) and women (Bischoff and Simpson, 2000), could be confirmed since more than half of the affected rhesus macaques (five of nine) were directly related to one breeding male. An underlying polygenetic inheritance is thereby assumed, which means that the disease is caused by the cumulative effect of several different genes acting in concert with first degree relatives holding more of the susceptibility genes than the average population (Bischoff and Simpson, 2000). Although the incidence of endometriosis is generally higher in nulliparous females (Schindler, 2007), which is also reported for the rhesus macaque, e.g., with 78 % in a cohort study of 72 animals (Fanton et al., 1986), all animals with spontaneous endometriosis from the investigated breeding colony had multiple offspring in the past. However, the time span between last birth and death was long (4–11 years) in all of them, and four animals had a history of abortion or stillbirth, indicating a disease-related impairment of fertility. Evidence for infertility associated with endometriosis is also described in several animal studies as well as human patients (reviewed in Giudice and Kao, 2004). As shown, for example, by data from IVF (in vitro fertilization) treatment, this is mainly caused by poor ovarian reserve in advanced disease, low oocyte quality, impaired embryo survival, and poor implantation capacity, most probably due to adverse effects of peritoneal fluid containing high concentrations of cytokines, growth factors, and activated macrophages in patients with endometriosis (Barnhart et al., 2002; Olivennes, 2003; Taketani et al., 1992).

The course of the disease and clinical symptoms with endometriosis are highly variable and closely associated with the nature and distribution of lesions (Fanton et al., 1986; Mounsey et al., 2006). According to the literature, three different types of endometriosis are recognized in humans: peritoneal endometriosis, deep infiltrating endometriosis, and ovarian cysts (also named endometriomas; Adamson, 2011; Young et al., 2013), and it is assumed that at least the pathogenesis for ovarian endometriosis might be different from that of peritoneal endometriosis (Brosens and Brosens, 2000a; D'Hooghe and Debrock, 2002). All three forms were also detected in the rhesus macaques of the present study; however, a clear separation between animals could not be confirmed. The major common macroscopic feature, found in eight of nine cases, corresponds to peritoneal endometriosis, characterized by fibrous adhesions and variable peritoneal plaque formation, at least comprising the pelvic organs. Ovarian cysts and large cystic masses were additionally present in two or three animals, respectively. And, as shown by histology, there was also more or less deep infiltration of endometriotic tissue present in all monkeys, regardless of the organ or tissue involved. This overlap of the three different endometriosis forms suggests at least some commonalities in pathogenesis. Together with the fact that the pelvic peritoneum is also the most common site for endometriotic lesions in over 80 % of human patients (Mahmood and Templeton, 1991), a crucial role of the peritoneum in the pathogenesis of endometriosis is obvious. In a recent review (Young et al., 2013), different peritoneal factors were highlighted that significantly influence the development and progression of endometriosis, emphasizing the implantation theory as the main pathogenetic mechanism. The authors suggest that the establishment of endometriosis in the peritoneal cavity requires refluxed endometrial tissue or cells, which are only able to adhere, infiltrate, and proliferate in cooperation with certain altered properties of the peritoneum. These alterations refer to mesothelial expression of adhesion molecules (e.g., cadherins and integrins) and changes in mesothelial cell morphology facilitating ectopic cell attachment and invasion together with tissue remodeling via matrix metalloproteinases and epithelial–mesenchymal transition (EMT). Furthermore, enhanced secretion of pro-inflammatory cytokines by activated peritoneal macrophages and mesothelial cells as well as immune evasion by impaired clearance mechanisms are assumed to support the establishment of endometriotic lesions, together with concurrent peritoneal cell proliferation and differentiation (for further detail, see Young et al., 2013).

In the present study, various histological features of endometriotic lesions were identified, both in terms of epithelial differentiation and the stromal phenotype ranging from cell-rich endometrial to collagen-rich fibrotic or loosely arranged myxoid forms to spindle cells with pronounced myogenic (SMA+) or angiogenic (vWF+) differentiation. This could be explained by induced EMT (as described above) and proliferation of either peritoneal multipotent mesenchymal stem cells or by endometrial mesenchymal stem-like cells that have been demonstrated in menstrual fluid, extrauterine endometrial implants (Figueira et al., 2011; Gotte et al., 2011; Matsuzaki and Darcha, 2012), and normal endometrium, respectively (Gargett et al., 2016). However, both ways most likely depend on the microenvironment and chronicity of the lesion. Additionally, in normal endometrium, it is recognized that the microenvironment determines the phenotype of endometrial tissue: active cyclic metaplasia of endometrial stromal cells (ESCs) into myofibroblasts and vice versa is observed in basal layers, whereas in superficial layers hormone-dependent secretion, vascularization, or bleeding is pronounced (Brosens and Brosens, 2000a). Accordingly, atypical or less differentiated forms of pure stromal endometriosis in rhesus macaques could easily be mistaken for spindle cell proliferation of other origin, such as retroperitoneal fibromatosis (RF), leiomyoma, inflammatory myofibroblastic tumors, inflammatory pseudotumors, or peripheral nerve sheath tumor (Bielefeldt-Ohmann et al., 2005). Thus, a careful evaluation of histological specimens in several sections should be performed for identification of possible glandular or cystic structures. And, if applicable, immunohistochemistry for epithelial markers (e.g., CK) should be carried out in cases of equivocal, more or less blood-filled cavities for discrimination between endothelial lining and flattened epithelium of an endometriotic cyst.

To the authors' knowledge, this is the first study that systematically compares the presence, distribution, and immunohistochemical characteristics of variable histological grades of differentiation in spontaneous endometriosis, both in human and nonhuman primates. The fact that all histological types were found more or less next to each other, or even merged, within the same animal strongly suggests parallel events of sequential differentiation processes. Furthermore, less differentiated histological grades, especially purely stromal forms but also the flattened epithelial components of endometriosis with mixed or poor differentiation, were sometimes difficult to distinguish from serosal tissue. Together with the demonstrated immunohistochemical co-expression of epithelial and mesenchymal markers (CK, vimentin, sometimes together with SMA and desmin), most obvious in poorly differentiated endometriosis and resembling distinct mesothelial cell properties, an induced differentiation of peritoneal cells into endometrial tissue, is conceivable, supporting the theory of coelomic metaplasia. The consistently reported presence of pluripotent mesenchymal stem cells (MSCs) in peritoneal tissue (Carmona et al., 2011; Mutsaers et al., 2015) could therefore serve as an essential prerequisite. Moreover, peritoneal mesothelial cells provide such a high degree of plasticity that, if placed in the appropriate microenvironment, they have the potential to generate various other mesenchymal-derived cell types (Gotloib et al., 2007). Hence, it is assumed that the different histological grades represent a subsequent graduation of differentiation in the course of time, with poorly differentiated types representing newly formed, immature lesions and well-differentiated types being older, fully differentiated forms, rather than being the outcome of dedifferentiation processes. The possible underlying mechanism of induced mesothelial differentiation into endometrial tissue (epithelial and stromal) might be exerted by direct cell-to-cell communication, probably via exosomes containing epigenetic regulatory molecules (e.g., miRNAs). Recent insights on the communication of tumor cells with adjacent mesenchymal tissue support this hypothesis (Webber et al., 2015). Moreover, exosomes isolated from endometrial stromal cells were capable of inducing angiogenic effects in human umbilical vein cells, pointing out that exosomes derived from ESCs play paracrine roles in the development of endometriosis (Harp et al., 2016). Thus, exosomes might work as effective intercellular communication modulators in endometriosis, exerting direct epigenetic effects on surrounding tissue, so that the endometriotic cells themselves do not need to “invade” the environment and further proliferate but rather stimulate the adjacent cells (e.g., MSCs) to become one of their kind. This assumption would also be supported by the present finding that proliferative activity in less differentiated grades of endometriosis was comparatively low.

In one of the two animals (no. 3) with conspicuous spindle cell proliferation in addition to endometriotic lesions, the exact cell origin is ambiguous. The histological appearance of streams and interwoven bundles of plump pleomorphic spindle cells within up to moderate amounts of collagenous matrix most likely resembles low grade fibrosarcoma, although retroperitoneal fibromatosis or other intestinal stromal tumors can also have a similar morphology (Bielefeldt-Ohmann et al., 2005). RF has been described in macaques with simian acquired immunodeficiency syndrome (SAIDS) associated with simian retrovirus type D (SRV-2) infection (Marx and Lowenstine, 1987), and is presumably caused by a gammaherpesvirus (retroperitoneal fibromatosis herpesvirus, RFHV; Bielefeldt-Ohmann et al., 2005; Rose et al., 1997). Lesions are typically restricted to the ileocaecal junction, mesenteric root, and mesenteric lymph nodes, and tumor cells are usually immunohistochemically positive for vimentin, vWF, and SMA (Fikes and O'Sullivan, 1995). However, the viral status for SRV-2 and RFHV of this rhesus monkey was not known and the pleomorphic spindle cells were positive for CK, vimentin, SMA, and desmin, suggesting an additional epithelial phenotype as also observed in the undifferentiated forms of concurrent endometriosis. Therefore, MSCs from endometrial or peritoneal tissue, being capable of pluripotent differentiation characterized by immunohistochemical expression of more than two histogenetically unrelated antigens, could have been the cell of origin in this case. A similar immunohistochemical expression pattern is also described for certain forms of mesothelioma (Fassina et al., 2012), but calretinin, a diagnostic marker for mesothelioma, has not been tested in the present case, so the definite diagnosis remains speculative.

The other spindle cell tumor (animal no. 6) that was found in the present study was consistent with leiomyoma and occurred together with multifocal adenomyosis, which is defined as the presence of ectopic endometrium within the myometrium. Besides in women, adenomyosis has been reported in several nonhuman primates and rarely other non-primate species (Barrier et al., 2007; Baskin et al., 2002; Greaves and White, 2006; Wilkinson et al., 2008). It is frequently accompanied by surrounding myometrial hyperplasia or concurrent leiomyoma (Barrier et al., 2007; Wilkinson et al., 2008). Some authors consider adenomyosis as a different form of endometriosis, and its pathogenesis is likewise obscure (Brosens and Brosens, 2000a).

One rhesus macaque (no. 4) revealed an intrauterine mass histologically confirmed as endometrial hyperplasia. Endometrial hyperplasia or polyps are reported in perimenopausal women or older nonhuman primate females, often associated with endometriosis, and can be induced by unopposed estrogen (Baskin et al., 2002; Bennett et al., 2009). Another striking endometrial change, caused by an increase in progesterone levels or induced by mechanical or chemical irritation (Marston et al., 1971), is decidualization of the endometrial stroma, an essential feature of the implantation stage of pregnancy in rhesus macaques and other primates (Beck et al., 2014). It involves stromal proliferation and decidual differentiation, characterized by large polyhedral cells with abundant faintly eosinophilic cytoplasm, distinct cell borders, and a centrally located, large, round to ovoid nucleus, often with a prominent nucleolus. Binucleation is regularly present, and the cytoplasm reveals a strong PAS-positive reaction due to high glycogen contents (Wadsworth et al., 1980). However, also ectopic decidualization or “deciduosis”, with groups of decidual cells located on serosal surfaces outside the uterus, occurs in the vast majority of pregnant women, usually as an incidental finding that regresses postpartum within 4 to 6 weeks, but sometimes leading to spontaneous fatal intraperitoneal hemorrhage (Kinra et al., 2006; O'Leary, 2006). Deciduosis can occur concurrently with endometriosis, as reported for macaques (Atkins et al., 2016; Beck et al., 2014) and women (O'Leary, 2006), and has also been detected in two animals of this study to varying extent. While in animal no. 3 only few small foci of decidual cells were found admixed with endometriotic tissue in the mesometrium, the other case (no. 6) revealed widespread decidualization of expansively growing endometriotic stroma located on the uterus, mesometrium, and urinary bladder. The same animal, being the only one of the examined population, underwent abdominal surgery 3 months prior to death and had a history of premature delivery with retained placenta. This suggests either surgically derived irritation or hormonal imbalances as possible causes for decidualization. Furthermore, decidual-like cells can also arise from mesothelial tissue independently of pregnancy or endometriosis, as known from rarely described deciduoid mesothelioma in human patients with predilection to the peritoneum and female preponderance in contrast to other types of mesothelioma (Shia et al., 2002).

Endometriosis is a hormone-dependent disorder, which is often treated with contraceptive steroids, progestagens, agonists of gonadotropin-releasing hormone (GnRH), and androgens in order to limit further growth and activity of ectopic endometriotic tissue (Giudice and Kao, 2004; Mounsey et al., 2006). Accordingly, the immunohistochemical staining of steroid receptors is a typically reported feature of endometriotic tissue in different species (Assaf and Miller, 2012; Fazleabas et al., 2003; Slayden and Brenner, 2004) and was also evident in the present study. However, the overall hormonal receptor scores in epithelial cells were considerably higher in normal endometrium and well-differentiated endometriotic lesions than in less differentiated forms. Furthermore, the level of hormonal receptor scores apparently correlated with the percentage of epithelial cells positive for Ki67, which has also been described by others (Toki and Nakayama, 2000), and, thus, a hormone-dependent proliferative activity of endometriotic glandular tissue, like in normal endometrium, is indicated. Nonetheless, previous analysis of Ki67 staining patterns in human endometriotic tissue compared to uterine endometrium revealed significantly reduced proliferative activity in endometriotic epithelium (Scotti et al., 2000). Though data on concurrent expression of steroid receptors and information about histological differentiation of endometriotic specimens were not provided, rather less differentiated forms of endometriosis were examined in that study, as is recognizable from the published pictures.

The other cell-cycle-related antigen tested in the present study was p53, also known as tumor protein TP53 or “guardian of the genome”, here showing consistent positivity in epithelial cells of endometriotic lesions with mixed or poor differentiation. As an important regulator of the cell cycle, e.g., by inducing cell-cycle arrest, activating DNA repair mechanisms, or initiating apoptosis, p53 becomes activated in response to numerous stressors, including but not limited to DNA damage, as well as oxidative stress or dysregulated oncogene expression (Surget et al., 2013). Inactivation of p53 by mutation or other genetic alterations leads to loss of function and can cause uncontrolled cell proliferation (Burns et al., 1991). Correspondingly, p53 overexpression has been detected in a wide variety of human malignancies, including ovarian endometrioid carcinoma (Harlozinska et al., 1996). Therefore, nuclear accumulations of mutated p53 protein are often supposed as a carcinogenic change. Accordant to our results, an overexpression of p53 has also been recognized in epithelial cells of several human endometriotic lesions (Toki and Nakayama, 2000). However, in those lesions neither mutations nor microsatellite alterations could be detected, so that the p53 accumulations are regarded as wild type, and, thus, a role other than oncogenic is considered. Additionally, the fact that the immunohistochemical p53 staining in our study did not correspond to PIs of the different histological grades indicates that the constantly detected accumulations of p53 in mixed or poorly differentiated epithelium are not related to uncontrolled cell proliferation.

Altogether, the results of the present study point out the diagnostic challenges of endometriosis in nonhuman primates related to clinical, macroscopic, and histological findings and provide novel insights into possible pathogenetic mechanisms derived from morphological characteristics, emphasizing the hypothesis of coelomic metaplasia. However, further research is necessary in order to verify the underlying mechanism of induced peritoneal differentiation into endometriotic tissue, especially regarding cell-to-cell communication via exosomes, which might potentially reveal new therapeutic approaches.

## Data Availability

All relevant data are presented in the paper. Please contact the corresponding author for further details.
